# Effect of Pingchuan Formula on Toll-Like Receptors and Dendritic Cells in an Asthmatic Mouse Model

**DOI:** 10.1155/2020/7407016

**Published:** 2020-08-31

**Authors:** Fei Liu, Li Bai, Zheng Xue, Wei Pan, Jianer Yu

**Affiliations:** ^1^Wuxi Hospital Affiliated to Nanjing University of Chinese Medicine, China; ^2^Shanghai Municipal Hospital of Traditional Chinese Medicine, Shanghai University of Traditional Chinese Medicine, Shanghai 200071, China

## Abstract

Pingchuan formula (PCF) was created by Professor Yu Jianer. The purpose of this study was to investigate the effect of PCF on dendritic cells (DCs) and toll-like receptors (TLRs) in initiating immunity. A bronchial asthma BALB/c mouse model was established using an OVA excitation method. PCF was immediately administered by gavage after the first excitation. After 7 d, hematoxylin and eosin (HE) staining was used to observe the pathological changes in the asthma model. Eosinophil infiltration and concentrations of IL-4, IFN-r, IL-12, and IFN-*α* in BALF were determined by enzyme-linked immunosorbent assay (ELISA). Real-time PCR was used to determine mRNA levels of IL-12 and IFN-*α*. Protein expression levels of ERK, Toll-2, IDO, and Toll-9 were measured by immunoblot. HE and ELISA showed that PCF could improve lung pathological changes and significantly decrease the concentration of IL-4 in BALF. Moreover, PCF could increase IL-12, IFN-*α*, and IFN-r in BALF. Real-time PCR and western blot showed that PCF restored the DCs and TLRs in initiating immunity. In summary, this study found that PCF can improve the pathological changes and reduce the symptoms of asthma in a BALB/c mouse model. It can facilitate the initiation of immunity by restoring the DCs and TLRs.

## 1. Introduction

Asthma is a common lung disease in childhood, which is associated with phlegm and wheezing. The prevalence of asthma is currently increasing worldwide at a rate of 50% every decade, and the number of asthma patients will reach 400 million by 2025 [1]. According to the statistics of the World Health Organization, there are more than seven million asthmatic children under the age of 18 years in the United States, and the economic burden due to asthma exceeds that of tuberculosis and AIDS combined [2]. Given the increasing incidence of asthma and its associated heavy economic burden, investigating its pathogenesis and effective treatment has become research hotspots worldwide.

With the research progress, various pathogenesis and treatment methods of asthma have been discovered. Although several treatment methods have been well established in western medicine, the problems of repeated attacks of wheezing and the long-term existence of phlegm remain. Hence, the application of traditional Chinese medicine is critical.

**Figure 1:**
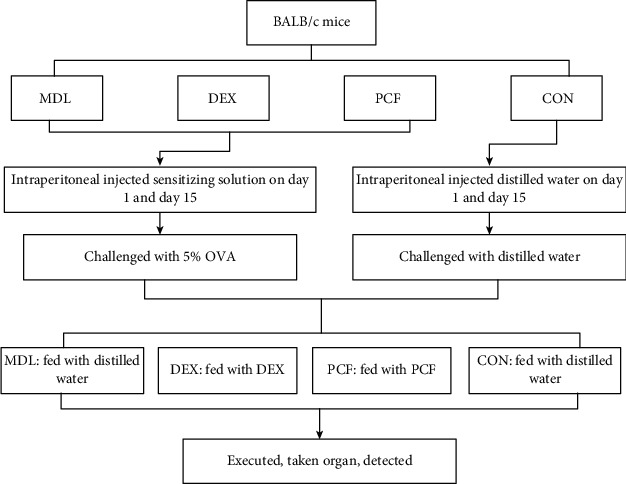
Experimental design.

Pingchuan formula (PCF) is a special herbal prescription for asthma that was created by Professor Yu Jianer. PCF is composed of roasted ephedra, bitter almond, purple Perilla, peach kernel, radish, prickly ash, dried earthworm, Scutellaria, and roasted licorice. The formula can modulate the QI movement in the lung, clear the phlegm, and remove the blood stasis, thereby relieving asthma. Immune mechanism has become the classic pathogenesis of asthma. PCF could significantly improve the imbalance of Th1/Th2 in a mouse model [3] and thereby modify the immune imbalance of asthma. It can also restore Treg/Th17 balance [4] and improve airway inflammation and airway reconstruction. The mechanism by which PCF regulates T cell differentiation remains unknown. The current study was performed to investigate the effect of PCF on the initiation of immunity. Moreover, the Toll-2/Toll-9/DC signal transduction pathway was detected in asthmatic mice (Figure 1).

## 2. Materials and Methods

### 2.1. Animals

A total of 40 male BALB/c mice were provided by Shanghai Super B&K Laboratory Animal Corp. Ltd. (grade SPF, 4-6 weeks old, 18-22 g in weight, production license number: SCXK2013-0016). The mice were housed in the animal experimental center of Shanghai Traditional Chinese Medicine University (room temperature: 25°C, humidity: 60-70%). All mice were provided free access to water and food.

### 2.2. Mouse Grouping

Forty mice were randomly divided into four groups: control group (CON, *n* = 10), model group (MDL, *n* = 10), dexamethasone group (DEX, *n* = 10), and PCF group (PCF, *n* = 10). The mice in the MDL group were not treated after modeling. The DEX and PCF groups were gavaged with DEX and PCF for seven days after modeling.

### 2.3. Model Establishment

Based on previously published modeling methods, combined with established modeling conditions and doses explored by our research group in the preliminary stage, the mouse asthma model was established. First, the mice received intraperitoneal (ip) injection of sensitizing solution on day 1 and day 15, except the CON group. The sensitizer consisted of 10 g OVA (Shanghai Jun Hui Chemical Co.), 1 g aluminum hydroxide, and 100 ml distilled water. Next, 24 hours after the last sensitization, the sensitized groups were challenged with 5% OVA (100 ml water containing 5g OVA) atomization excitation, for 40 min each time, once a day for one week. The CON group were ip injected and inhaled an equal volume of distilled water.

### 2.4. Administration of Drugs by Gavage

PCF (provided by Shanghai Hospital of TCM) was prepared at 5.33 g/ml concentration as previously described [3]. Different concentrations of PCF were previously tested by us, and this concentration was found to be the most appropriate [5]. Dexamethasone (Shanghai Xinyi Co., Ltd.) was prepared at a concentration of 0.075 mg/ml. Mice in the CON and MDL groups were fed with distilled water. According to the formula in the pharmacology of traditional Chinese Medicine, all groups were fed with 20 ml/kg.

### 2.5. Eosinophil Infiltration in BALF

Mice were sacrificed, the chest was opened, and the right main bronchi was clamped with hemostatic forceps. A No. 12 blunt needle was inserted into the left main bronchus and then douched two times with 1 ml phosphate-buffered saline (PBS) at 4°C [French PBS buffer solution (pH 7.2-7.4)]. BALF was centrifuged (Germany Eppendorf centrifuge Model: 5804R), and eosinophils (EOS) were assayed using an ELISA kit as per the manufacturer's instructions (mouse Eotaxin ELISA kit RD, USA).

### 2.6. Determination of Cytokine Profiles in BALF

The concentrations of IL-12, IL-4, IFN-r, and IFN-*α* in BALF were measured using sandwich enzyme-linked immunosorbent assay (ELISA) kits (R&D Systems, Minneapolis, MN, USA) according to the manufacturer's instructions.

### 2.7. HE Staining of Lung Tissue

Paraffin-embedded lung sections (Microtome Leica RM2235, 5 *μ*m thick) were stained with HE, and the lung structure was evaluated by an experienced pathologist using a fluorescence microscope (Olympus BX51, Tokyo, Japan).

### 2.8. Real-Time PCR Assay of Lung Tissue

Lung sections (0.05 g) were cut and placed into a Ribozyme test tube to extract RNA, followed by amplification of the prepared cDNA. The total reaction volume was 50 *μ*L, comprising of real-time PCR Mix 32 *μ*L, IL-12, IFN-*α*R 0.5 *μ*L, TaqMan probe 0.5 *μ*L, ddH_2_O 14.5 *μ*L, and cDNA template 2 *μ*L. Reaction conditions were as follows: 95°C for 5 min, 95°C for 15 s, and 60°C for 45 s, 40 cycles. Using beta-actin as an internal reference, the relative gene expression was calculated by a 2^-*ΔΔ*Ct^ method.

### 2.9. Immunoblot Assay

Lung fragments (0.05 g) were homogenized in ice-cold RIPA lysis buffer (Beyotime, China), centrifuged at 14,000 rpm for 15 min, and the supernatant was collected. Protein concentrations were determined by the BCA protein assay kit (Beyotime, China). Polyacrylamide gel (10%) electrophoresis was used to isolate the proteins. The proteins were then transferred to a nitrocellulose membrane (Bio-Rad, Hercules, CA). The nitrocellulose membrane was probed with anti-Toll-2, anti-ERK, anti-Toll-9, or anti-IDO monoclonal antibodies at 4°C overnight. The next day, the membrane was incubated with horseradish peroxidase-labelled goat anti-rabbit IgG antibody (1 : 1000) at room temperature, followed by alkaline phosphatase-conjugated secondary antibodies (BD, USA) for 2 h at room temperature on a shaker. The protein bands were displayed by ECL™ western blotting detection (GE healthcare, No. RPN2106) and quantified by Gel-Pro imaging software.

## 3. Statistics

Statistical analysis was conducted using SPSS 18.0 software. If the data followed a normal distribution and homogeneity of variance, a one-way analysis of variance (ANOVA) and least significant difference (LSD) multiple comparison were used. Otherwise, the data were converted. A *P* value < 0.05 was considered significant.

## 4. Results

### 4.1. Comparison of Symptoms among the Groups

The mice in the MDL group showed clustering phenomenon, different degrees of anxiety, muscle twitching, urinary and fecal incontinence, hair fluffing, lower activity, appetite reduction, irritability, and other symptoms. The DEX and PCF groups were better.

### 4.2. Comparison of Pathological Changes among the Groups

**Figure 2:**
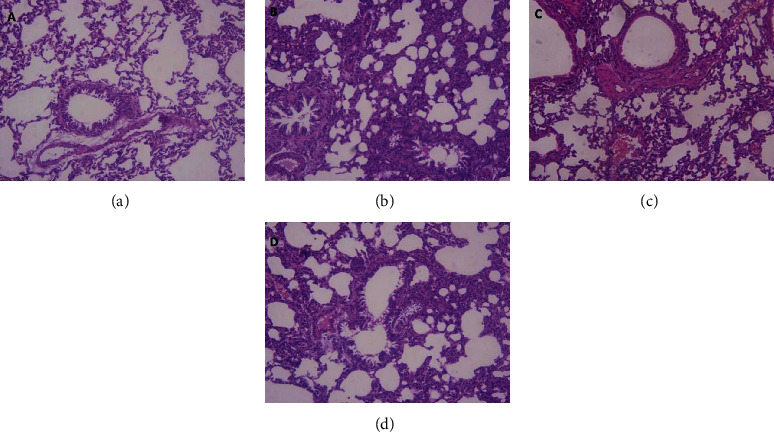
Pathological changes detected using HE staining. (a) CON group, (b) MDL group, (c) DEX group, and (d) PCF group.

Mice in the CON group were normal. The bronchial structure was smooth and intact. The cells were arranged in neat rows, and no obvious abnormality was found in the lumen and peritubule. In the MDL group, the bronchus was deformed, the diameter of the bronchus was narrowed, and the wall structure was damaged, with extensive inflammatory exudates in the lumen and numerous inflammatory cell infiltration and accumulation in the perivasculature. The DEX and PCF groups were better than the MDL group. They showed bronchial structure integrity. The cells were disordered but cell diameter was normal. There was some inflammatory cell infiltration and accumulation in the perivasculature (Figure 2).

### 4.3. The Eotaxin Content in BALF

**Figure 3:**
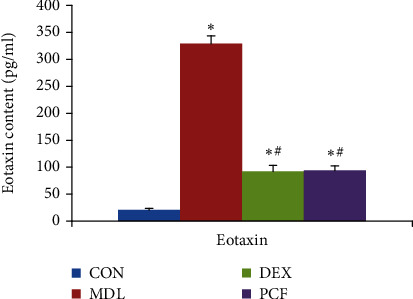
Eotaxin content in BALF: ^∗^compared with the CON group, *P* < 0.05; ^#^compared with the MDL group, *P* < 0.05.

The mice in the MDL group had extremely high levels of Eotaxin as compared to the CON group. In contrast, the DEX and PCF groups had reduced Eotaxin in BALF (Figure 3).

### 4.4. Comparison of Toll-2/Toll-9/DC Cytokines IL-12, IL-4, IFN-r, and IFN-*α* among the Groups

**Figure 4:**
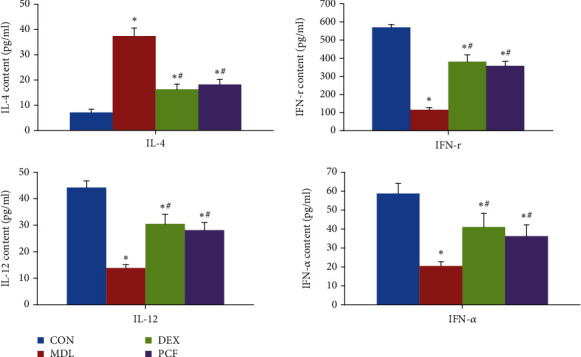
Comparison of Toll-2/Toll-9/DC cytokines IL-12, IL-4, IFN-r, and IFN-*α* among the groups. ^∗^Compared with the CON group, *P* < 0.05; ^#^compared with the MDL group, *P* < 0.05.

As shown in Figure 4, the MDL group had higher levels of IL-4 than the CON, DEX, and PCF groups, but IL-12, IFN-r, and IFN-*α* levels were lower in the MDL group (*P* < 0.05). There was no statistically significant difference between the PCF and DEX groups.

### 4.5. Comparison of IL-12 and IFN-*α* Gene Expressions among the Groups

**Figure 5:**
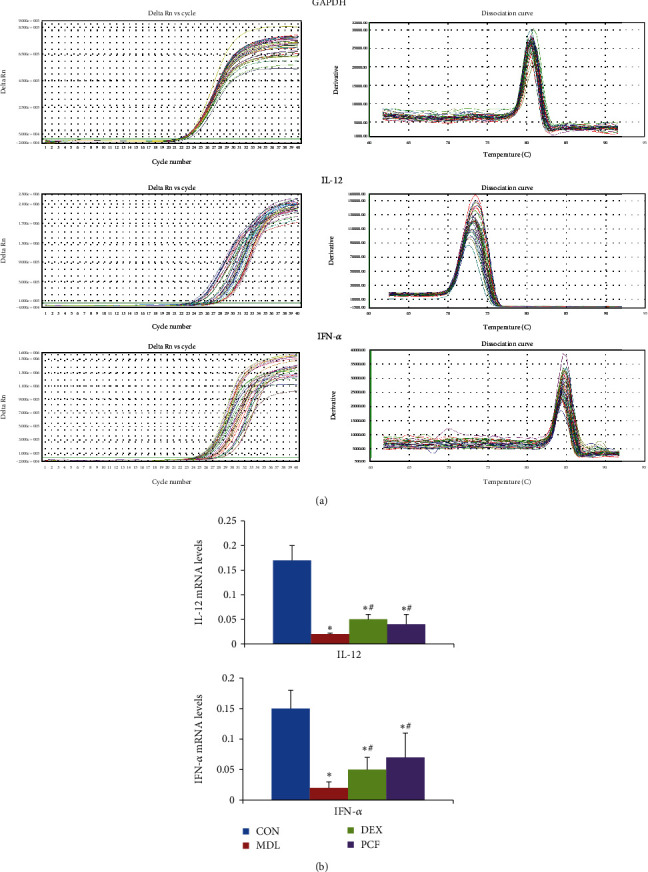
Comparison of IL-12and IFN-*α* gene expressions among the groups. (a) Amplification kinetics and melting curves of IL-12 and IFN-*α* in the lung tissues of the CON, MDL, DEX, and PCF groups; (b) quantitative analysis of (a); ^∗^compared with the CON group, *P* < 0.05; ^#^compared with the MDL group, *P* < 0.05.

The data showed that the MDL group had lower levels of all cytokines than the CON, DEX, and PCF groups (*P* < 0.05, Figure 5). The PCF and DEX groups could improve the molecular expression, but the differences between them were not stably significant.

### 4.6. Expression of ERK, Toll-2, Toll-9, and IDO Proteins in Asthmatic Airways

**Figure 6:**
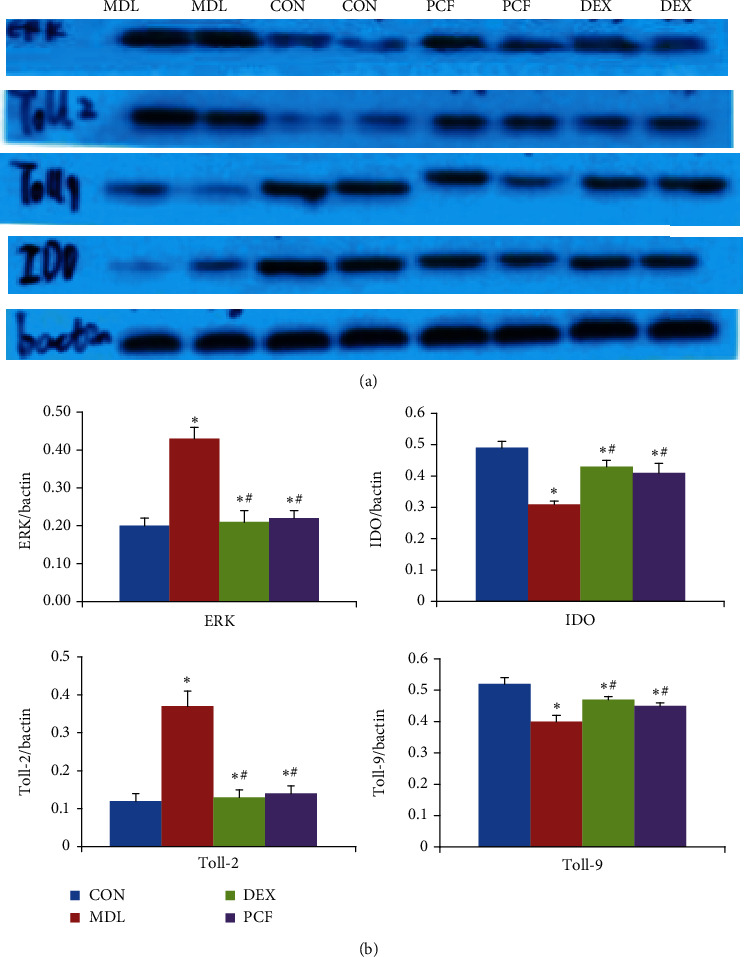
Expression of ERK, Toll-2, IDO, and Toll-9 proteins in asthmatic airways. (a) Immunohistochemistry assay of ERK, Toll-2, IDO, and Toll-9 in lung tissues of the CON, MDL, DEX, and PCF groups; (b) quantitative analysis of (a); ^∗^compared with the CON group, *P* < 0.05; ^#^compared with the MDL group, *P* < 0.05.

As shown in Figure 6, ERK and Toll-2 expression was higher in the MDL group than in the CON group, but Toll-9 and IDO were lower in the MDL group (*P* < 0.05). The DEX and PCF groups had lower ERK and Toll-2 but higher Toll-9 and IDO levels than the MDL group (*P* < 0.05). There was no significant difference between the DEX and PCF groups.

## 5. Discussion

Dendritic cells (DCs) are specialized antigen-presenting cells that are the main promoters of immune response. They activate unsensitized T cells participate in the differentiation and development of T cells and induce and maintain immune tolerance. Traditional DCs (cDCs) stimulate T primordial cells to Th2, differentiate Th17 cells, develop immune response, and promote airway inflammation. Plasmacytoid DCs (pDC) can stimulate the production of Treg cells and regulate pulmonary immune tolerance formation [6, 7]. However, immature DCs (imDC), low expression of MHC-II molecules, and lack of stimulation of T cells are needed. Consequently, DCs must be activated to initiate the activation phase of antigen-specific lymphocytes, which is accompanied by the production of costimulatory molecules and cytokines. The activation of these DCs is mediated by toll-like receptors (TLRs).

TLR is a pattern recognition receptor (PRR), which recognizes pathogen-associated molecular patterns (PAMP), activates the corresponding activation signal, participates in the activation of innate immunity and adaptive immunity, and establishes a bridge between innate immune response and adaptive immune response. TLR is mainly expressed on DCs and plays an important role in regulating the immune response of antigen-specific T cells. [8]. imDC cannot effectively present and process antigens. After ingesting antigens, the maturation and migration of DCs is initiated [9]. DCs recognize foreign antigens by TLR-PAMP. Therefore, TLR can affect the immune response by modulating the maturation of DCs.

Studies have shown that TLR-2, which is mainly expressed on cDCs, is the main TLR [10] involved in asthmatic airway inflammation. It can promote Th2 differentiation. Gene polymorphism is related to asthma development and the progression of lung function [11]. The activation of TLR-2 can induce high-intensity and lasting phosphorylation of extracellular regulated kinase (ERK), which prevents the production of IL-12p70, and induces the classical immune response of Th2 [12, 13]. Shibata et al. [14] found that the activation of the ERK signaling pathway determines the development of Th2-dependent eosinophilic inflammation and antigen-mediated airway hyperresponsiveness. Yamashita et al. [15] showed that the differentiation of Th2 depends on the activation level of the ERK signaling pathway.

TLR-9 can activate plasma-like DCs by recognizing CpG motifs of foreign antigens. pDC can secrete large amounts of IFN-*α* [16, 17], which is considered a necessary upstream factor to regulate the expression of IDO [18]. It can specifically induce the expression of IDO and promote the secretion of IFN-r. Studies have shown that IDO is an immune regulatory enzyme. Its regulatory mechanism is closely linked with the secretion of IFN. IDO-IFN determines the formation of immune tolerance [19, 20]. IDO can promote the production of Treg cells, which are crucial immunoregulatory cells capable of suppressing Th1 and Th2-mediated adaptive immune responses in a cell contact-dependent fashion. Moreover, the secretion of IFN-r can stimulate the production of Th1.

In this study, MDL was a successful model, with extensive intraluminal inflammatory infiltration, perivascular inflammatory cell infiltration, and accumulation. There was more eosinophil infiltration than in the CON group. The level of IL-4 in BALF was higher in the MDL group than in the CON group, and the levels of IL-12, IFN-*α*, and IFN-r in BALF were lower than in the CON group. The gene expression of IL-12 and IFN-*α* was lower in the MDL group than in the other groups. The expression of ERK, Toll-2, IDO, and Toll-9 was disturbed, with an increase in ERK and Toll-2 and a decrease in IDO and Toll-9. These differences were statistically significant compared with the CON group.

This study revealed that PCF can downregulate ERK and Toll-2 and upregulate IDO and Toll-9 expression, reduce IL-4 level, and increase IL-12, IFN-*α*, and IFN-r in BALF, thus restoring the balance of T cells via TLRs and DCs to improve airway inflammation and reduce asthma symptoms. All indexes were statistically significant. Therefore, PCF can regulate the differentiation of T cells by participating in the initiation of immunity.

The current study had several limitations. The mechanism of TCM treatment is very complex. However, this study uncovered the novel role of PCF in initiating the immune response. It would be beneficial to determine the effectiveness of PCF in the long-term treatment of asthma.

## Data Availability

The data used and analyzed in the figures of this study are available from the corresponding author on reasonable request.

## Abbreviations

PCF: Pingchuan formula
DCs: Dendritic cells
TLRs: Toll-like receptors
HE: Hematoxylin and eosin
ELISA: Enzyme-linked immunosorbent assay
CON: Control group
MDL: Model group
DEX: Dexamethasone group
ip: Intraperitoneal
PBS: Phosphate-buffered saline
EOS: Eosinophil
ANOVA: Analysis of variance
cDCs: Traditional DCs
pDC: Plasmacytoid DCs
imDC: Immature DCs
PRR: Pattern recognition receptor
PAMP: Pathogen-associated molecular patterns
ERK: Extracellular regulated kinase.
